# Neutrophil reverse migration

**DOI:** 10.1186/s12950-022-00320-z

**Published:** 2022-11-24

**Authors:** Qichao Xu, Wenqi Zhao, Mingyang Yan, Hongxia Mei

**Affiliations:** 1 Department of Anesthesia and Critical Care, The Second Affiliated Hospital and Yuying Children’s Hospital of Wenzhou Medical University, 109 Xueyuan Road, Wenzhou, Zhejiang Province People’s Republic of China 325027; 2 Key Laboratory of Anesthesiology of Zhejiang Province, The Second Affiliated Hospital and Yuying Children’s Hospital of Wenzhou Medical University, Zhejiang, China

**Keywords:** Neutrophil reverse migration, Inflammation, Neutrophils

## Abstract

The behavior of neutrophils is very important for the resolution of inflammation and tissue repair. People have used advanced imaging techniques to observe the phenomenon of neutrophils leaving the injured or inflammatory site and migrating back into blood vessels in transgenic zebrafish and mice, which is called neutrophil reverse migration. Numerous studies have shown that neutrophil reverse migration is a double-edged sword. On the one hand, neutrophil reverse migration can promote the resolution of local inflammation by accelerating the clearance of neutrophils from local wounds. On the other hand, neutrophils re-enter the circulatory system may lead to the spread of systemic inflammation. Therefore, accurate regulation of neutrophil reverse migration is of great significance for the treatment of various neutrophil- mediated diseases. However, the mechanism of neutrophil reverse migration and its relationship with inflammation resolution is unknown. In this review, we reviewed the relevant knowledge of neutrophil reverse migration to elucidate the potential mechanisms and factors influencing reverse migration and its impact on inflammation in different disease processes.

## Introduction

Neutrophils are the main component of leukocytes, accounting for 50 -70% of the total number of leukocytes and play an essential role in anti-infection. Neutrophils are known to be the first cells to reach the site of inflammation and their chemotaxis to the site of inflammation is the critical immune response process in the management of tissue infection and injury. Neutrophils have complex functions. They can eliminate pathogens through phagocytosis, degranulation, reactive oxygen species (ROS), and neutrophil extracellular traps (NETs) [1–3]. However, the excessive accumulation or persistence of neutrophils at the site of inflammation can delay inflammation resolution, develop into chronic inflammation, and ultimately hinder tissue repair [2]. Therefore, removal of neutrophils from the wound site can help to reduce inflammation and avoiding tissue damage [4]. Traditionally, it was believed that the main pathway of clearance of neutrophil after they have completed their task is local apoptosis at the site of inflammation and subsequent phagocytosis by macrophages [5]. However, with the development of imaging techniques, Mathias et al.in 2006 observed for the first time the reverse migration of zebrafish neutrophils from the site of inflammation back into the vascular system and introduced the concept of “reverse neutrophil migration”, suggesting that reverse neutrophil migration is also an important pathway to clear activated neutrophils [6]. Subsequently, the same phenomenon was observed in a mouse model [7]. In 2017, Wang et al. visualized the reverse migration of neutrophils in mice, and studied in depth the reverse migration of neutrophils in a model of aseptic liver heat injury, confirming that: reverse migration of neutrophils occurs into two phases, the first phase is the return of neutrophils from the site of inflammation to the blood vessels, and the second phase is the reverse migration of neutrophils from the blood vessels back to the bone marrow to promote apoptosis [8]. In this case, the process of neutrophil re-entry from the site of inflammation to the vascular lumen is also known as reverse transendothelial migration (rTEM). There is currently more research on rTEM.

What are the effects of reverse migration of neutrophils on the organism? Some scholars believe that reverse migration regulates the resolution stage of inflammation, reduces neutrophils retention at the inflammatory site, and accelerates the resolution of local inflammation [6]. However, some researchers have also found that the return of neutrophils from the wound site to the blood vessel may contribute to the spread of systemic inflammation [9]. The phenomenon of reverse migration indicates that neutrophils may have potential novel functions. Accurate control of neutrophil reverse migration may be an important therapeutic strategy to promote inflammation resolution. We review the current research progress of neutrophil reverse migration to provide new ideas for applying neutrophil reverse migration in the treatment of inflammatory.

## Reverse migration of neutrophils

The form of neutrophil movement has attracted much attention. At the initial stage of inflammation, many chemokines will be released from the wound site [5], and neutrophils are recruited to play a role in the inflammatory site along the chemotactic gradient of the chemokines, while neutrophils leaving the wound at the site of inflammation may be accomplished by a inverse chemotactic gradient. Some researchers have analyzed the reverse migration of neutrophils through mathematical modeling and believed that reverse migration is a process of random redistribution. They noted that reverse migration is caused by desensitization of neutrophils to chemotactic signals rather than rejection of chemical signals [10, 11]. The most direct evidence of this view is the in vitro U-shaped microfluidic model. In this model, neutrophils are first affected by a chemoattractant and moved along the chemotactic gradient. After a period of time, we find that most human neutrophils start to move away from the chemoattractant. The retrograde cells were more than 90%, and the movement distance was more than 1000 μm [12]. This indicates that neutrophils can desensitize to chemotactic signals and reverse chemotactic gradient movement. The movement form of neutrophil drifting and diffusion not only occurs in zebrafish fin injury, mouse ischemia-reperfusion injury, and human neutrophils in vitro experiments, but also occurs in a variety of injuries. For example, in zebrafish experiments with laser damage to the heart, neutrophils in the pericardial region spread to the dorsal and posterior parts of the injury site, so they are far away from the injury site. They also spread randomly in their motile form to adjacent tissues without entering specific organs [13]. Heart injury and fin injury have similar immune cell dynamics during the neutrophil immune response, but the immune response of the heart injury model seems to be more ‘moderate. ‘.

Some scholars believe that neutrophils leave the inflammatory site due to the influence of chemotactic signals far away from the inflammatory site. Recent studies have shown that reverse migration is related to vascular permeability. When inflammation occurs, the vascular permeability increases, and the chemokines released by tissues and immune cells diffuse int the blood vessel wall. Endothelial cells bind to some of these chemokines and present them in the lumen of the vessel, resulting in a greater concentration of certain chemokines in the plasma than at the injury site, thus prompting neutrophils to migrate into the vessels. During the reverse migration phase, an increase in the concentration of CXCL1 in the blood can be detected, and the increased concentration of CXCL1 is sufficient to drive the reverse migration of neutrophils [14].

Neutrophils have a variety of reverse migration patterns [15]. One pattern is reverse transendothelial migration, whereby neutrophils can ‘crawl ‘from the wound to the vascular endothelium, cross the endothelial cells into the blood circulation, and spread to other parts of the body. Neutrophils can also leave the wound in other ways, such as metastasis from adjacent tissues [15, 16] and lymphatic metastasis [17], and eventually spread to other parts of the body.

Compared with neutrophils in resident tissues and circulation, neutrophils undergoing reverse migration have a unique phenotypes. The neutrophil phenotype after reverse migration was cell adhesion molecule-1 (ICAM-1 or CD54) ^high^, CXCR1^low^, while neutrophils in resident tissue were CD54^low^, CXCR1^low^, and the circulating neutrophils were CD54^low^, CXCR1^high^ [18]. The expression of ICAM-1 mRNA in the lung and thymus of septic mice was significantly increased [19], indicating the presence of reverse migrating neutrophils in the lung and thymus tissues of septic mice, which also suggests that the vulnerability of infected patients to acute lung injury may be related to reverse migration of neutrophils. Neutrophils have multiple phenotypes in the body, which means that different phenotypes of neutrophils may play different functions. Although the phenotype of neutrophils differs before and after reverse migration, zebrafish studies showed that neutrophils’ response and antibacterial effect after reverse migration on secondary injury were not significantly different from those at the recruitment stage [20].

Neutrophils reverse migration of neutrophils has been widely studied in the past decade, and there are many different views on this phenomenon. We still need more detailed and more convincing evidence to accurately explain the phenomenon of neutrophil reverse migration. At the same time, we also need to master many aspects of reverse migration, such as mechanism, influencing factors, and drug targets.

## Possible mechanism of neutrophil reverse migration

The specific mechanism of neutrophils reverse migration is still unclear, so neutrophils have been observed to migrate from wounds through surrounding tissues [7, 8, 16] or reverse transendothelial migration [6, 21] using genetic markers and advanced imaging techniques. Here we describe some potential mechanisms.

### The mechanism of adhesion molecule C(JAM-C) at the junction of endothelial cells

Reverse transendothelial migration was first observed in real-time imaging transgenic zebrafish models [6]. Neutrophils can successfully break through the endothelium and return to the circulatory system, indicating that there may be a channel on the endothelium. When this channel is open, neutrophils enter the circulatory system through this channel. The ‘switch ‘of this channel is the junctional adhesion molecule JAM-C. In the ischemia-reperfusion (I-R) injury model of the mouse levator muscle, the expression of JAM-C at the junction of mouse vascular endothelial cells was decreased after injury, which was blocked by pharmacological methods [21]. Breaking off JAM-C on endothelial cells or knockdown of the JAM-C gene significantly increase the probability of neutrophil reverse migration, which is mainly achieved through the paracellular pathway [21]. In addition, an increase in reverse migration was also observed after JAM-C gene knockout in mice with acute pancreatitis. This evidence indicates that JAM-C is an essential factor in the regulation of reverse migration. It is difficult for neutrophils that migrate back into the circulation after reverse migration to migrate back to the inflammatory site [18], indicating that the reverse migration channel associated with JAM-C is a unidirectional channel. Reverse transendothelial migration was common in ischemia-reperfusion injury [21]. We speculate that JAM-C is an important factor in the construction of vascular integrity. Ischemia-reperfusion injury leads to the destruction of vascular integrity. At this time, neutrophils successfully enter the circulation from the ‘gap ‘at the vascular endothelial junction under the induction of a specific signal. Similar phenomena also occurred in monocytes, and JAM-C also regulated the rTEM of monocytes [22]. Therefore, JAM-C plays a crucial role in mediating leukocyte migration, not just neutrophils.

**Fig. 1:**
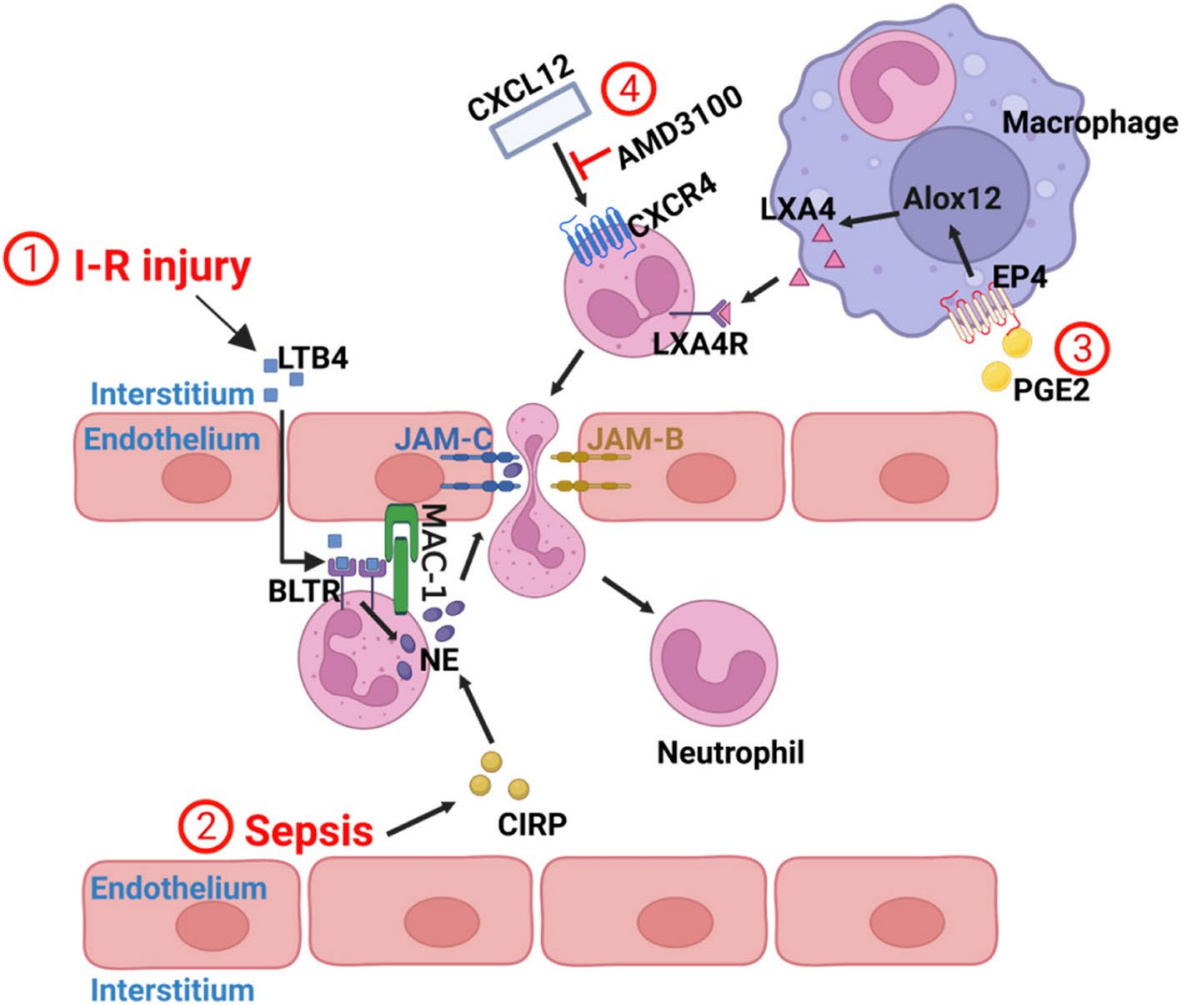
① I-R injury stimulates the production of LTB4, which binds to BLTR receptors on intravascular neutrophils to induce NE secretion. Neutrophils then transfer NE to EC-JAMC via MAC-1 and hydrolyze it, causing neutrophils at the site of injury to return to the bloodstream through this damaged “gap”. ② In sepsis, CIRP expression is increased and promotes neutrophil producing NE, which subsequently destroys EC-JAMC through the mechanism described in ①. ③ PE2 can signal through the EP4 receptor of macrophages, resulting in increased production of the Alox12 gene, thereby promoting endogenous LXA4 production. LXA4 acts on neutrophils at the injured site and promotes rTEM. ④ Increased neutrophil reverse migration following inhibition of CXCL12/CXCR4 signaling on neutrophils using AMD3100

Why is the expression of JAM-C on endothelial cells decrease during an ischemia-reperfusion injury? It is related to the leukotriene B4-Neutrophil Elastase Axis. Nourshargh et al. confirmed that the LTB4 - NE axis is a promoter of reverse transendothelial migration: expression of the lipid chemotactic agent LTB4 was increased during ischemia-reperfusion injury in the cremaster muscle of mice. LTB4 binded to BLTR on intravascular neutrophils and stimulated neutrophils to produce excess neutrophil elastase (NE), then neutrophils delivered NE to EC-JAMC via Mac-1, and finally NE hydrolyzed endothelial JAM-C protein, resulting in loss of JAM-C as shown by ① in Fig. 1 [23]. The results suggest that local LTB4-NE axis is a promoter of neutrophil rTEM and provide evidence that this pathway can propagate a local sterile inflammatory response into a systemic inflammatory response.

In addition, in some studies, we have found that the LTB4-NE axis is not only related to rTEM, but is also closely linked to secondary organ damage. It was found that intradermal injection of LTB4 into the cremaster, buccal muscle or ear skin of mice resulted in neutrophils accumulation in the lungs and distal organ damage (such as heart, liver, etc.) [23]. We know that NE inhibitor GW311616A does not affect neutrophil recruitment [23]. Injection of LTB4 into the buccal muscles of mice pretreated with GW311616A resulted in a significant reduction in neutrophils in the lungs of mice compared to mice injected with LTB4 alone [23]. In another experiment, the neutrophil chemoattractant KC and NE were injected simultaneously into the cremaster muscle of mice [23]. More neutrophils entered and infiltrated in the lungs than when KC or NE were injected alone. When KC and NE were simultaneously injected into the buccal muscle of EC JAM-C-specific deletion mice, significant pulmonary edema wasinduced [23]. In conclusion, these studies suggest that the LTB4-NE axis promotes neutrophil reverse migration and leads to secondary organ damage.

Studies have found that extracellular cold-inducible RNA-binding protein (CIRP) may induce the reverse migration of septic neutrophils by upregulating NE expression and down-regulating JAM-C expression in mouse lungs [24]. Sepsis was induced by cecal ligation and puncture (CLP) in male C57BL / 6 mice, and it was found that the frequency of neutrophil reverse migration in the blood after CLP treatment increased in a time-dependent manner, reaching the highest frequency after 20 hours [24]. In the absence of CIRP, even 20 hours after cecal ligation and puncture,the frequency of reverse migration was significantly reduced in mice. rmCIRP treatment for 5 hours resulted in a significant increase in lung NE and a decrease in JAM-C expression in mice. When CIRP was removed,the results were reversed. However, neutrophils reverse migration was not entirely dependent on CIRP, as JAM-C expression was only increased by 35% in CIRP-deficient mice compared to wild-type mice [24]. The presence of CIRP provides a new possibility for the treatment of acute lung injury in patients with sepsis. CIRP may be related to the LTB4 - NE axis as shown by ② in Fig. 1, but the relationship between them and the mechanism of CIRP-induced NE increase remains to be further investigated. This evidence also indicates that excessive NE may be essential in promoting neutrophil reverse migration.

### HIF signaling pathway mechanism

In addition, studies in zebrafish have shown that the HIF signaling pathway is associated with neutrophils reverse migration. HIF is a transcription factor that acts on a variety of immune cells, such as macrophages, T cells, B cells and neutrophils and plays an important role in the immune and inflammatory response of the body [25]. The HIF signaling pathway regulates the resolution period of inflammation and the HIF-1 path is closely related to the survival and movement of neutrophils. HIF-1α can inhibit the apoptosis of neutrophils under hypoxia conditions [26]. In zebrafish experiments, activated HIF-1α not only inhibits apoptosis, but also reduces neutrophils reverse migration, thereby delaying inflammation resolution [16]. However, because the HIF-1 pathway can act on multiple immune cells and has distinct functions in different cells, we can’t determine whether the reduction in reverse migration is caused by the direct effect of HIF-1α on neutrophils or by HIF-1α affecting other immune cells,which in turn affect neutrophils. The reduction of reverse migration after HIF-1α activation may be partly responsible for the slower resolution of inflammation, but this still needs to be proved in mammalian experiments.

### Lipid mediating mechanism

The presence of lipid mediators has also been associated with neutrophils reverse migration. Prostaglandin E2 (PGE2) is one of the critical cell growth factors and regulators. PGE2 is the most abundant prostaglandin in the body and has multiple effects on inflammation [27]. It was found that the transverse injury of zebrafish caudal fin could induce the production of PGE2. PGE2 could signal through the EP4 receptor to increase the production of the ALOX12 gene, thereby promoting the production of endogenous lipoxin A4 (LXA4), enhancing the reverse migration of neutrophils at the site of trauma and ultimately promoting the resolution of local inflammation, as shown by ③ in Fig. 1 [7]. In this experiment, the reverse migration speed and movement path of PGE2-treated meutrophils were not significantly different from those of the control group, indicating that PGE2 did not enhance reverse migration by accelerating neutrophils movement speed, but rather promoted neutrophils to leave the wound site [7]. LXA4 inhibits neutrophil chemotaxis in vitro [28, 29], which may be one of the reasons for reverse migration. The promotion of neutrophil reverse migration by PGE2 and LXA4 provides a potential therapeutic target for inflammatory diseases, but the exact mechanisms by which they regulate neutrophil migration and promote inflammation resolution is still unclear and requires further investigation.

### CXCL12/CXCR4 signal axis mechanism

Reducing the excessive retention of neutrophils at the site of inflammatory is critical to promote inflammation resolution. CXCL12 / CXCR4 signal axis retains neutrophils at the site of inflammation [11]. Renshaw et al. demonstrated that inhibiting CXCL12 / CXCR4 signal axis could accelerate the deterioration of local inflammation by knocking down the expression and pharmacological inhibition of CXCR4b and CXCL12a in zebrafish through CRISPR / Cas9 gene-editing technology [11]. Under the treatment of AMD3100, a drug that inhibits CXCL12 / CXCR4 signal, neutrophil reverse migration is increased and the resolution of local inflammation is accelerated as shown by ④ in Fig. 1 [11]. It indicates that it is possible to treat inflammation by inhibiting the signal axis through a pharmacological methods. In addition, CXCR2 / CXCL8 signaling pathway regulates neutrophil reverse migration [30–32]. Inhibition of this signaling pathway can reduce reverse migration, for example by using CXCR1 / 2 selective inhibitor (SB225002) [30, 33, 34]. In this pathway, neutrophils are repulsive to CXCL8 in vitro [35]. Nevertheless, in the experiment of Renshaw et al., the results were different. In their experiments, reverse migration occurred when neutrophils were pretreated with low concentrations of CXCL8 and then exposed to high concentrations of CXCL8 [32]. Neutrophils at high concentration but without pretreatment showed high chemotaxis in CXCL8, while chemotaxis was significantly lower in neutrophils at the same concentration of CXCL8 but pre-activated. The form of movement of these low chemotaxis neutrophils resembled random motion, and they migrated with increased distance and speed [32]. These phenomena suggest that neutrophils not only reject CXCL8 but also appear to be insensitive to chemokines.

### Macrophages promote neutrophil reverse migration

Macrophages also play a specific role in promoting the reverse migration of neutrophils. Macrophages control neutrophils reverse migration through redox and Src family kinase signals, which are achieved by contacting neutrophils [36]. Huttenlocher et al. found that 57% of neutrophils in zebrafish larvae containing macrophages underwent reverse migrated 2 h after injury. In comparison, only 35% of neutrophils migrated reversely in the irf8 mutant larvae with macrophages defects. Moreover, neutrophils spent longer at the site of injured and left the wound at a slower and shorter direction [36]. Reverse migration of neutrophils and positive interaction between neutrophils and microglia have also found in central nervous inflammation [37]. However, the direct contact between macrophages and neutrophils is not essential to mediate neutrophil reverse migration, as zebrafish larvae may still undergo reverse migration when macrophages are absent [13, 36]. In another experiment, the researchers observed that 71% of the reversely migrated neutrophils did not directly contact with macrophages and 65% of the reversely migrated neutrophils directly contacted with macrophages [7]. The tracking analysis of macrophages and neutrophils motility trajectories before and after direct contact also showed no significant change in neutrophil migration behaviour after direct contact [7]. These indications indicate that the promotion of neutrophils reverse migration by macrophages can be achieved not only be through direct contact, but also that substances secreted by macrophages may indirectly affect neutrophils.

To date, the exact mechanism of neutrophil reverse migration is unclear.

## Drugs that affect neutrophil reverse migration

The effect of some anti-inflammatory drugs is linked to the regulation of neutrophil reverse migration. If we could control neutrophils reverse migration through drugs, it would be greatly beneficial to improve the patients’ condition. Here we have listed some medications associated with reverse migration to provide a new idea for the treatment of inflammation.

Tanshinone IIA is a Chinese herbal medicine with anti-inflammatory activity. The mechanism of tanshinone IIA accelerating inflammation in vivo is related to neutrophils. Some scholars evaluated the effect of tanshinone IIA on freshly isolated human neutrophils by morphology and Annexin V / PI staining and found a significant increase in apoptosis after 8 hours of incubation with tanshinone IIA in neutrophils [38]. However, in the zebrafish caudal fin injury model, where neutrophil apoptosis is low, tanshinone IIA accelerated inflammation resolution by promoting neutrophil reverse migration [38]. In addition, tanshinone IIA can also inhibit the role of HIF-1α and promote tissue repair [38] even in the presence of HIF activation signal (which, as mentioned above, is considered to delay inflammation resolution). Loss of Cftr would lead to the continuous accumulation of neutrophils at the site of inflammatory. Tanshinone IIA reverses this phenomenon and reduces inflammatory injury in cystic fibrosis (CF) zebrafish [39]. We speculate that the mechanism of tanshinone IIA driving inflammation resolution in humans may also be related to the promotion of reverse neutrophil migration. Although no increase in tanshinone IIA-induced reverse migration was observed when human neutrophils were analysed using an in vitro model of endothelial monolayer reverse migration [38], this may be due to the difference between the in vivo and in vitro environments. Whether tanshinone IIA can promote the neutrophils reverse migration in vivo requires further experiments in humans or other mammals. The question of how tanshinone IIA acts on neutrophils deserves further investigation.

Kuding tea is a kind of tea rich in chlorogenic acid, which has anti-inflammatory and anticancer effects. Recent studies have found that chlorogenic acid-enriched extract of kuding tea promotes neutrophil reverse migration by inducing phosphorylation of ERK and AKT proteins. ERK [40] and AKT [41] are known to regulate neutrophil migration. In the zebrafish embryo experiment, treatment of Kuding tea extract reduced cell apoptosis and increased phosphorylation levels of ERK and AKT proteins at the site of injured (Western blot analysis) [42]. These phenomena suggest that Kuding tea chlorogenic acid extract may exert its anti-inflammatory mechanism by promoting neutrophil reverse migration.

The relative levels of different lipid mediators can also promote the resolution of inflammation and may be involved in the reverse migration of neutrophils. As mentioned previously, LTB4 is a neutrophil chemoattractant, but following a switch in lipid mediator class, the pro-inflammatory lipid mediator production pathway is altered in favour of pro-regressive mediators such as lipotoxins, abasicins, and protectins [43, 44]. They can block the influx of new neutrophils [45]. They are also associated with promoting the resolution of existing neutrophil inflammation. LXA4’s appearance in microfluidic device in vitro indicates that LXA4 can enhance reverse migration of human neutrophils.

## Effects of neutrophil reverse migration on different diseases

Many studies in mice and zebrafish have shown that reverse migration reduces the accumulation of neutrophils at the site of trauma and accelerates local inflammation resolution. However, neutrophils migrating back into the blood vessels may also lead to the spread of systemic inflammatory. That is to say, reverse migration is a double-edged sword. How to balance the resolution of local inflammation and the spread of systemic inflammation is a crucial issue for us. Here we explain the effects of reverse migration in different animal disease models to further understand reverse migration.

### Reverse migration promotes local inflammation resolution

In the zebrafish larvae caudal fin or ventral fin aseptic injury model, zebrafish neutrophils can be directly observed leaving the wound and migrating back into the blood vessels [6, 16, 23, 46]. The number of reverse migration neutrophils exceeds 80% [6]. When the HIF-1α gene was knocked out [16] or when Kuding tea extract [42] and tanshinone IIA [38], etc. were added to promote neutrophils reverse migration, the experimental results showed that the tissue repair of fin injury was accelerated. The effect is reversed if the frequency of neutrophil reverse migration is reduced. In addition to fin damage, the same results were also observed in zebrafish heart damage model. Cardiac injury includes low-temperature freezing injury [34] and laser injury [13]. In the zebrafish heart cryopreservation model, the researchers added CXCR1 / 2 selective inhibitor SB225002, which could inhibit neutrophils reverse migration (as described above) and retain many neutrophils at the site of wound. The experimental results showed that treatment of SB225002 accelerated the hematologic reconstitution at the site of cardiac injury, but SB225002 significantly reduced the number of cardiomyocytes and inhibited the cardiomyocyte regeneration [34], indicating that reducing neutrophils reverse migration hinders the regeneration of wound tissue.

Reverse migration is also exist in mice during the recovery from acute renal ischemia-reperfusion injury. After acute renal ischemia-reperfusion injury, ICAM-1^high^neutrophils with THP (a kidney-specific protein) [47] were detected in the blood circulation, suggesting that cells undergo reverse migration. Reducing JAM-C expression resulted in a significant increase in the number of ICAM-1^high^ neutrophils with THP in the blood and accelerated recovery renal function and tissue repair. It was confirmed that reverse migration accelerated the resolution of local inflammation.

### Neutrophil reverse migration causes systemic inflammatory diffusion

Neutrophils reverse migration also leads to the spread of pathogenic neutrophils to distal organs, thereby promoting the spread of systemic inflammation. We have previously explained that reverse migration is associated with the expression of JAM-C, which also plays a massive role in promoting the spread of systemic inflammation. In mice suffering from testicular I-R injury [21, 23] and acute pancreatitis [5], we found that reducing reduced JAM-C expression led to increase neutrophils reflux to pulmonary vessels, thereby aggravating the inflammation of the lungs inflammation. Acute pancreatitis mice with JAM-C gene knockout developed severe acute lung injury and systemic inflammation, with a higher proportion of neutrophils in peripheral blood and pulmonary vessels of JAM-C deficiency mice than in control mice [9]. In addition, acute lung injury caused by sepsis was also related to reverse migration [24]. In clinical practice, patients with sepsis are prone to induce acute lung injury (ALI) [48], and results of lung injury caused by reverse cross transendothelial migration of neutrophils have also been obtained in a mouse model of sepsis [24].

If the conclusion that reverse migration causes the spread of systemic inflammation is true, then blocking reverse migration could reduce the spread of systemic inflammation. We have previously explained that the LTB4-NE axis is related to reverse migration. In RA and LPS-induced acute pancreatitis mice, acute pancreatitis leads to an increase in LTB4 and its specific receptor BLT1, which aggravates the lung injury associated with acute pancreatitis [49]. At the same time, the percentage of neutrophils with CD54^high^ and CXCR1^low^phenotypes in peripheral blood increased significantly, indicating that acute pancreatitis is closely related to neutrophils reverse migration. After BLT1 blockade by LY293111, the number of neutrophils returning to circulation was reduced, the percentage of neutrophils with the unique phenotypes of CD54 ^high^ and CXCR1^low^ in peripheral blood decreased and the degree of lung injury was reduced [50]. These evidence shows that LTB4 aggravates acute pancreatitis-related lung injury by promoting neutrophil reverse migration. In addition, we know that neutrophil elastase has a positive effect on reverse migration, and Japanese hospitals use the enzyme inhibitor to reduce postoperative pulmonary inflammation [51] and to treat patients with acute lung injury complicated with systemic inflammatory response syndrome [50], indicating that the effect of neutrophil elastase inhibitor may be related to blocking reverse migration.

Why does reverse migration lead to secondary organ damage and the spread of systemic inflammatory? The septic thermal liver injury experiment in mice [13] shows the motion path of neutrophils back into the circulation. After neutrophil recruitment, the majority of the neutrophils at the site of injury will return to the vascular system. The number of neutrophils at the site of injury peaks 12 h after injury. 24 h later, the number of neutrophils decreases by more than 90%. 48 h later, they almost disappeared and only about 10% of neutrophils undergo apoptosis [8]. Neutrophils migrating back into blood vessels from the injury site are not randomly distributed to various organs or tissues in the body as we predicted, and they will enter the bone marrow and lungs through a predetermined path [8]. GFP ^+^ neutrophils with reverse migration markers were detected in bone marrow and lung, and CXCR4 was upregulated in the lung. Interestingly, treatment with CXCR4 antagonist AMD3100 resulted in increased GFP ^+^ neutrophils in the lungs and decreased GFP ^+^ neutrophils in the bone marrow of mice. It is known that CXCR4 upregulation of neutrophils allows them to return to bone marrow via CXCL12 [52]. This phenomenon suggests that neutrophils first move to the lungs and then migrate selectively to the bone marrow through CXCR4 [8]. Although acute lung injury was not found in liver injury mice, it may be due to the fact that the extent and nature of this injury is different from that of I-R injury. Neutrophils in the lungs do increase temporarily and may develop into acute lung injury. We know that aging neutrophils return to bone marrow and are then phagocytized by macrophages [53]. The destination of neutrophils after reverse migration appears to be bone marrow. In addition to the return of reverse migrating neutrophils to bone marrow after liver injury, some studies have also found that neutrophils can transport a modified vaccinia virus Ankara strain to bone marrow through reverse transendothelial migration [54].

It is well known that activated neutrophils are prone to produce reactive oxygen species(ROS) and that excessive ROS can lead to tissue or organ damage. After reverse migration, neutrophils produce a large amount of ROS [18, 21]. These cells accumulate in the lungs or other organs, which can easily cause tissue damage. This may be one of the reasons for the spread of systemic inflammation due to neutrophil reverse migration.

In general, reverse migration is positive for local inflammation resolution, but neutrophils returning to the circulation can spread to secondary organs, causing secondary organ damage and leading to the spread of systemic inflammation. In particular, in infectious inflammatory diseases, the migration of large numbers of activated neutrophils back into the blood vessels can easily cause the spread of inflammation in distal organs, especially those with an abundant blood supply, such as the lungs, and it is therefore particularly important to rapidly upregulate the expression of CXCR4 on post-migrating neutrophils to reduce their residence time in the lungs and return them to the bone marrow for apoptosis.

## Future perspectives

In recent decades, greater grogress has been made in our understanding of neutrophils with the help of in vivo imaging techniques and genetic technologies. However, neutrophil reverse migration remains a phenomenon to be investigated. While excessive and prolonged neutrophil infiltration can lead to the development of chronic tissue inflammation, the migration of neutrophils back into the circulation may lead to systemic inflammation and tissue damage in distal organs. It is likely that the answer to this question depends on the nature, magnitude, and location of the inflammation. In the case of infection, premature neutrophil reverse migration may result in failure to clear the infection and dissemination of intracellular pathogens to other sites. In the case of tissue injury, reverse migration might have positive effects at the local site of inflammation, as neutrophils depletion may promote wound healing, but may have negative effects systemically such as multiple organ failure. Therefore, by precisely controlling reverse migration, it is possible to improve the acute inflammation of patients and prevent the inflammation from developing into chronic inflammation and secondary organ damage. However, most of the reverse migration observed to date has occurred in zebrafish and mice, and the behavior of human neutrophils in vivo needs to be further studied.

Recent studies have shown that inflammation resolution is an actively programmed process regulated by the production of a series of endogenous anti-inflammatory and pro-regressive mediators [55]. The newly proposed “pro-inflammatory regression” strategy emphasizes the protective effect of an appropriate inflammatory response on the body, i.e. not only suppressing the excessive inflammatory response at the onset of inflammation (at the source), but also attempting to promote the resolution of inflammation, in which accelerating the timely clearance of neutrophils, enhancing the phagocytosis of macrophages and promoting tissue repair and regeneration are key. Crucially,pro-inflammatory mediators (SPM) are an important endogenous pro-inflammatory mediator produced during the process of inflammation regression. Combine with the previously described ability of SPM to promote neutrophils reverse migration and thus accelerate inflammation resolution, there is still much scope to explore the detailed mechanisms and optimisation of its pharmacological effects. With the in-depth study of reverse migration, it is expected to provide new therapeutic strategies for anti-infection research and innovative ideas for clinical anti-infection treatment.

## Data Availability

Not applicable.

## Abbreviations

ROS: reactive oxygen species
NETs: neutrophil extracellular traps
rTEM: reverse transendothelial migration
I-R: ischemia-reperfusion
NE: neutrophil elastase
CLP: cecal ligation and puncture
PGE2: Prostaglandin E2
LXA4: lipoxin A4
